# Parkin Levels Decrease in Fibroblasts With Progranulin (PGRN) Pathogenic Variants and in a Cellular Model of PGRN Deficiency

**DOI:** 10.3389/fnmol.2021.676478

**Published:** 2021-05-13

**Authors:** Katarzyna Gaweda-Walerych, Dawid Walerych, Mariusz Berdyński, Emanuele Buratti, Cezary Zekanowski

**Affiliations:** ^1^Laboratory of Neurogenetics, Mossakowski Medical Research Institute, Department of Neurodegenerative Disorders, Polish Academy of Sciences, Warsaw, Poland; ^2^Laboratory of Human Disease Multiomics, Mossakowski Medical Research Institute, Polish Academy of Sciences, Warsaw, Poland; ^3^Molecular Pathology Group, International Centre for Genetic Engineering and Biotechnology (ICGEB), Trieste, Italy

**Keywords:** frontotemporal lobar degeneration, progranulin deficiency, parkin, TDP-43, primary fibroblasts

## Abstract

Frontotemporal lobar degeneration (FTLD) and amyotrophic lateral sclerosis (ALS) are neurodegenerative diseases with TDP-43 mislocalization and aggregation. Genetic forms of FTLD and ALS are caused by pathogenic variants in various genes, such as *PGRN* (progranulin). To date, depletion of parkin E3 ubiquitin protein ligase, a key mitophagy regulator, has been reported in sporadic ALS patients and ALS mice models with TDP-43 proteinopathy. In this work, we show parkin downregulation also in fibroblasts derived from FTLD patients with four different PGRN pathogenic variants. We corroborate this finding in control fibroblasts upon PGRN silencing, demonstrating additionally the decrease of parkin downstream targets, mitofusin 2 (MFN2) and voltage dependent anion channel 1 (VDAC1). Importantly, we show that TDP-43 overexpression rescues PRKN levels upon transient PGRN silencing, but not in FTLD fibroblasts with PGRN pathogenic variants, despite upregulating PGRN levels in both cases. Further observation of PRKN downregulation upon TDP-43 silencing, suggests that TDP-43 loss-of-function contributes to PRKN decrease. Our results provide further evidence that parkin downregulation might be a common and systemic phenomenon in neurodegenerative diseases with TDP- 43 loss-of-function.

## Introduction

Mitochondrial dysfunction is one of the common mechanisms underlying the pathogenesis of many neurodegenerative diseases, including amyotrophic lateral sclerosis (ALS) and frontotemporal lobar degeneration (FTLD) (Wang et al., 2013; Onesto et al., 2016; Davis et al., 2018). The main neuropathological hallmark of sporadic and genetic forms of ALS and FTLD caused by pathogenic variants in PGRN and many other genes, is TDP-43 (transactive response DNA-binding protein 43 kDa) pathology. It is characterized by TDP-43 protein depletion from the nucleus and its cytoplasmic accumulation as ubiquitin-positive inclusions (Neumann et al., 2006). This leads to both toxic gain- and loss-of-TDP-43 function, causing deregulation of various aspects of RNA metabolism (splicing, RNA stability and transport, microRNA processing, etc.), and protein, and mitochondrial homeostasis (Buratti et al., 2010; Wang et al., 2013; Ratti and Buratti, 2016; Gao et al., 2019), which eventually results in neuronal degeneration.

Defective or old mitochondria are targeted for degradation ***via*** (a) the ubiquitin-proteasome system which marks mitochondrial outer membrane proteins for disposal, and (b) autophagy, which eliminates mitochondria as whole organelles through lysosomal pathway (Scheibye-Knudsen et al., 2015). Selective elimination of mitochondria ***via*** autophagic pathway is called mitophagy (Rugarli and Langer, 2012). Several seminal papers on mitophagy initiation have previously demonstrated that PINK1 (PTEN-induced kinase 1) recruits parkin to damaged mitochondria to prime selective mitophagy (Narendra et al., 2008, 2010; Matsuda et al., 2010), followed by PINK1/parkin—dependent ubiquitination of various mitochondrial outer membrane proteins, such as MFN2 (Mitofusin 2), Tomm20 (translocase of outer mitochondrial membrane 20), and VDAC1 (voltage dependent anion channel 1) (Glauser et al., 2011; Gaweda-Walerych and Zekanowski, 2013). It is worth noting that pathogenic variants in ***PARK2*** gene encoding parkin lead to a loss of parkin function causing recessive, early/juvenile onset Parkinson’s disease (PD) with mitochondrial dysfunction. Importantly, PD-related symptoms (parkinsonisms) are found in FTLD with PGRN pathogenic variants (FTLD-PGRN) (Pickrell and Youle, 2015; Rowe, 2019). On the other hand, biallelic ***PARK2*** pathogenic variants have been associated with the phenotype of behavioral FTLD (Zimmermann et al., 2018). Interestingly, apart from **PD**, decreased levels of parkin have been found in motor neurons with TDP-43 inclusions derived from the spinal cord of sporadic ALS patients (Lagier-Tourenne et al., 2012) and cellular and animal models of TDP-43 proteinopathy (Sun et al., 2018). The depletion of parkin and its downstream targets, MFN2 and MIRO1, has also been observed in mitochondria derived from mice bearing G93A ***SOD1*** mutation, a genetic model of ALS with TDP-43 proteinopathy (Palomo et al., 2018). To date, regulation of parkin mRNA and protein levels, or its cellular localization has been linked to TDP-43 complex neuropathology or manipulations of TDP-43 expression, yielding discordant results (see Supplementary Table 3; Polymenidou et al., 2011; Lagier-Tourenne et al., 2012; Hebron et al., 2013; Davis et al., 2018; Sun et al., 2018).

However, it is not known whether the TDP-43 pathology exerts similar effects in different genetic backgrounds, e.g., *SOD1* vs. *PGRN* pathogenic variants. Indeed, *PGRN* pathogenic variants, apart from leading to TDP-43 pathology in affected brain tissue, confer a particular landscape of molecular changes including, among others, deregulation of the lysosomal pathway (Tanaka et al., 2017; Paushter et al., 2018).

Here, we demonstrate for the first time that parkin is downregulated in fibroblasts derived from carriers of PGRN pathogenic variants with PGRN haploinsufficiency. We further corroborate this finding using control human fibroblasts upon *PGRN* silencing and show that *TDP-43* silencing or overexpression in the context of PGRN depletion can modulate parkin levels to some extent.

## Materials and Methods

### Ethical Issues

All subjects gave their informed consent for participation in this study. The study was conducted in accordance with the Declaration of Helsinki, and the protocol was approved by the Bioethical Committee of the Central Clinical Hospital of the Ministry of Interior Affairs and Administration in Warsaw and the Bioethical Committee of Medical University of Gdańsk.

### Cell Culture, Treatments, siRNA Silencing, and Transient Transfection

Human primary fibroblasts were obtained from skin biopsies of four neurologically healthy control subjects and six FTLD patients carrying four different *PGRN* pathogenic variants (M1L *n* = 1; A9D *n* = 1; Q337X *n* = 2; C26Sfs *n* = 2) (Supplementary Table 1). Two fibroblast lines (with M1L and A9D) were obtained through the NINDS Human Cell and Data Repository (NHCDR) at Infinity Biologix (formerly RUCDR; NHCDR IDs: ND42493 and ND40082, respectively). Fibroblasts were maintained in standard conditions (DMEM supplemented with 4.5 g glucose, 1% GlutaMAX, 10% FBS, 1% Penicillin/Streptomycin) or in galactose medium (glucose-free DMEM supplemented with 5 mM galactose (Sigma) instead of glucose). The usage of culture medium with galactose had the objective to force fibroblasts to rely more on aerobic respiration than on glycolysis. The absence of mycoplasma infection was confirmed with PCR Mycoplasma Detection Kit according to manufacturer’s instruction (TOKU-E). All experiments were conducted on cells with the same or similar passage number < 10.

For silencing, primary human skin fibroblasts were transfected with siRNA (Sigma-Aldrich) and Lipofectamine RNAiMAX (Thermofisher Scientific) as per the manufacturer’s instructions. siRNA used for *PGRN* silencing was predesigned (SASI_Hs01_00096296-992NM_002087, Sigma). The siRNA sequence (sense: 5′-GCAAAGCCAAGAUGAGCCU-3′) was used to silence human *TDP-43* (Mohagheghi et al., 2016), while a non-targeting siRNA (sense: 5′-CTATAACGGCGCTCGATAT-3′) was used as a control (Sigma). RNA and proteins were collected 48 or 72 h after siRNA transfection.

For overexpression experiments, 0.5–2 μg of plasmids (pEGFP, pFlag-TDP-43 wild-type (WT) or pFlag-TDP-43 F4L, a mutant unable to bind RNA) (Ayala et al., 2011) were transfected with Lipofectamine 2000 (Invitrogen) according to manufacturer protocol. Each transfection experiment was performed at least three times, and a representative result is shown. Proteins were collected 24 or 48 h after plasmid transfection.

### RNA Extraction, Reverse Transcription and Real Time PCR

RNA was extracted and reverse transcribed according to standard protocol with TriReagent (Ambion) and QuantiTect^®^ Reverse Transcription kit (Qiagen), respectively. Quantitative real time PCR analysis was done with RT HS-PCR Mix SYBR (A&A BIOTECHNOLOGY) (primers are listed in Supplementary Table 2), using a StepOne Plus system (Applied Biosystems). Changes in gene expression were determined with the ΔΔCt method. Normalization against housekeeping genes, such as cyclophilin B (*PPIB*) or *GAPDH* gave similar results.

### Western Blot

Cultured primary fibroblasts was lysed in Cell Lysis Buffer (Cell Signaling), and 10–30 μg of proteins in Laemmli buffer were resolved by SDS-PAGE and electro-transferred to PVDF membranes. Western blot (WB) assays were performed using antibodies specific for MFN2 (1:300), VDAC1 (1:300), FLAG (1:500), TDP-43 (1:300) and Beta-actin (1:4,000) (all monoclonal mouse from Santa Cruz Biotechnology), TDP-43 C-terminal (1:300, Proteintech, cat. no. 12892-1-AP), PGRN (1:1,000, Sigma), parkin (1:500, Prk8, mouse antibody, cat. no. 4211, Cell Signaling), and GAPDH (1:20,000, Millipore). Proteins on membranes were visualized by chemiluminescence reagent (Biorad) and autoradiography.

### Statistical Analysis

For each experiment the relative values obtained from different biological replicates (*n* ≥ 3) were used to calculate mean, standard deviations (SD), and statistical significance in two-tailed *t*-test (GraphPad Prism 6.0). *p* < 0.05 was considered significant. For quantitative densitometric analysis of WB results, ImageJ software was used (Schneider et al., 2012). Intensity value of each protein band was normalized to respective B-actin or GAPDH value.

## Results

### Decreased Parkin mRNA and Protein Levels in Carriers of PGRN Pathogenic Variants and Upon *PGRN* Silencing in Control Fibroblasts

**FIGURE 1:**
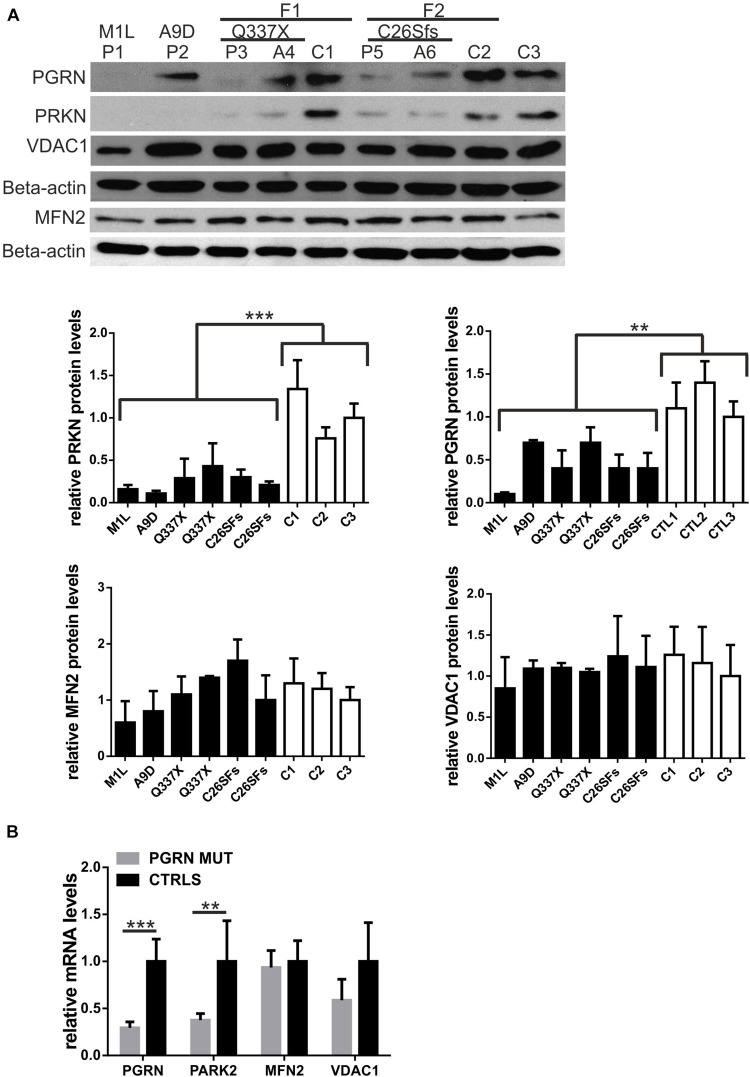
Parkin levels are downregulated in fibroblasts with PGRN pathogenic variants. **(A)** PRKN, PGRN, MFN2 and VDAC1 protein levels were determined by Western blot in fibroblasts with progranulin (PGRN) pathogenic variants and control ones. FTLD patients carrying PGRN pathogenic variants are designated as: P1 (M1L), P2 (A9D), P3 (Q337X), P5 (C26Sfs); asymptomatic young carriers of PGRN pathogenic variants are designated as: A4 (Q337X) and A6 (C26Sfs); neurologically healthy control subjects are designated as: C1, C2, and C3. Study participants P3, A4, and C1 belong to family 1 (F1); study participants P5, A6, and C2 subjects belong to family 2 (F2). Lower panels represent densitometric measurement of PRKN, PGRN, MFN2, and VDAC1 levels (normalized to Beta-actin levels). The results obtained for C3 were normalized to 1 and all the other results were normalized to C3; **p < 0.01;***p < 0.001. **(B)** PRKN (PARK2), PGRN, MFN2, and VDAC1 mRNA levels were determined by real-time PCR in fibroblasts with progranulin (PGRN) pathogenic variants (PGRN MUT: P1, P2, P3, P5, A4, and A6) and control ones (CTRLS: C1, C2, and C3). The results obtained for control lines were normalized to 1 **p < 0.01;***p < 0.001. PRKN, parkin RBR E3 ubiquitin protein ligase; PGRN, progranulin; MFN2, mitofusin 2, VDAC1, voltage dependent anion channel 1.

**FIGURE 2:**
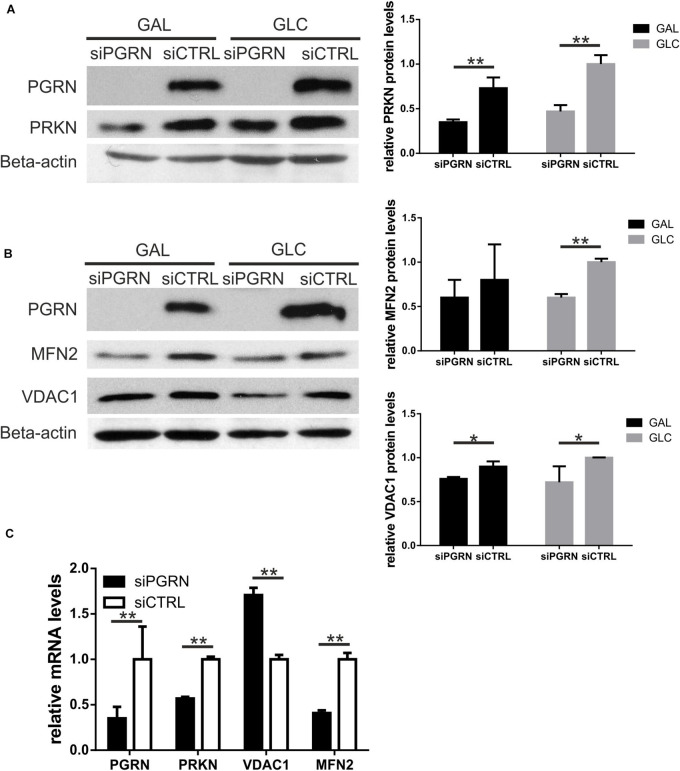
Parkin levels, along with MFN2 and VDAC1, are downregulated in control fibroblasts upon PGRN silencing. **(A)** PRKN and PGRN protein levels were determined by Western blot upon PGRN silencing for 48 h in control fibroblasts cultured in galactose (GAL) and glucose (GLC) medium; scramble siRNA-treated cells have been used as a control. PGRN levels were determined to evidence efficient PGRN silencing. Beta-actin was used as a protein load control. Right panel shows densitometric measurements of PRKN protein levels (normalized to Beta-actin level). Similar results were obtained upon normalization to GAPDH levels (not shown), **p < 0.01; **(B)** MFN2, and VDAC1 protein levels were determined by Western blot upon PGRN silencing for 48 h in control fibroblasts cultured in galactose (GAL) and glucose (GLC) medium; scramble siRNA-treated cells have been used as a control. Beta-actin was used as a protein load control. Right panel shows densitometric measurements of MFN2 and VDAC1 protein levels (normalized to Beta-actin level). Similar results were obtained upon normalization to GAPDH levels (not shown). *p < 0.05, **p < 0.01; **(C)** *PGRN, PRKN, MFN2*, and *VDAC1* mRNA levels were determined by real-time PCR upon PGRN silencing for 48 h in control fibroblasts cultured in glucose medium. For each gene measurement, scramble siRNA-treated cells have been used as a control and normalized to 1, **p < 0.01. PRKN, parkin; PGRN, progranulin; MFN2, mitofusin 2, VDAC1, voltage dependent anion channel 1.

Single PGRN pathogenic variants lead to decrease in *PGRN* mRNA and protein by c.a. 50–70% (Baker et al., 2006; Cruts et al., 2006). Thus, first, we confirmed lead to a decrease levels of PGRN protein and mRNA in all six carriers of PGRN pathogenic variants (Figures 1A,B), see also (Gaweda-Walerych et al., 2018). Then, we analyzed the mRNA and protein levels of a key mitophagy regulator—parkin (PRKN) and its downstream targets, VDAC1 and MFN2 in fibroblasts from six carriers of four different PGRN pathogenic variants (M1L, A9D, Q337X, and C26Sfs), compared to control fibroblasts. We observed decreased PRKN protein and mRNA levels, while MFN2 and VDAC1 levels were not significantly altered (Figures 1A,B). In order to further extend these observations, we created a genetic model of PGRN insufficiency where we silenced *PGRN* in primary skin fibroblasts from elderly, neurologically healthy control subject, C3 (Figures 2A–C). Since high glucose metabolism was shown to decrease parkin levels, which could be further exacerbated by PGRN deficiency (Zhou et al., 2019), we analyzed the effects of *PGRN* silencing both on glucose and galactose medium, obtaining similar results in both media (Figures 2A,B). As shown in this figure, we observed downregulation of parkin protein and mRNA (Figures 2A,C) after *PGRN* silencing for 48 h, accompanied by concordant changes in MFN2 protein and mRNA (Figures 2B,C), and in the level of VDAC1 protein. In contrast, VDAC1 mRNA level increased (Figure 2C). Moreover, to further support these conclusions we observed that increasing doses of *PGRN* siRNA led to efficient *PGRN* silencing and PRKN downregulation on protein and mRNA level (Supplementary Figures 1A,B), with effects persisting at 72 h (Supplementary Figure 1A), whilst silencing of PGRN in other control subject (C4) led to PRKN downregulation as well (Supplementary Figure 1C).

### TDP-43 Overexpression and Silencing in PGRN Mutation Carriers

The 25 kDa fragment of TDP-43 C-terminal cleavage is a major component of insoluble cytoplasmic aggregates observed in affected brain regions of FTLD-PGRN patients and in cellular models with stably silenced *PGRN* (Neumann et al., 2006; Zhu et al., 2019). As expected, we confirmed an increased production of a 25 kDa TDP-43 C-terminal cleavage product in control fibroblasts with silenced *PGRN*, compared to scrambled control siRNA (Supplementary Figure 2A). Furthermore, we observed increased production of a 25 kDa C-terminal product in fibroblast line derived from FTLD patient (P3) with PGRN pathogenic variant Q337X, compared to control fibroblast line (C3) (Supplementary Figure 2B). These results indicate the increased 25 kDa production as a hallmark of TDP-43 pathology in fibroblast with PGRN mutation and following transient *PGRN* silencing.

TDP-43 has been reported to regulate *PGRN* mRNA stability (Colombrita et al., 2012). In particular, TDP-43 overexpression was shown to increase PGRN levels through sortilin 1 (SORT1) receptor upregulation (Prudencio et al., 2012). In parallel, it has been demonstrated that manipulations of TDP-43 level (overexpression or silencing) affected parkin mRNA and protein levels (Polymenidou et al., 2011; Lagier-Tourenne et al., 2012; Davis et al., 2018; Sun et al., 2018; Supplementary Table 3). To validate these results in our experimental models of PGRN deficiency, we either silenced or transiently over-expressed TDP-43 in fibroblasts with silenced *PGRN* and in fibroblasts with PGRN pathogenic variants.

**FIGURE 3:**
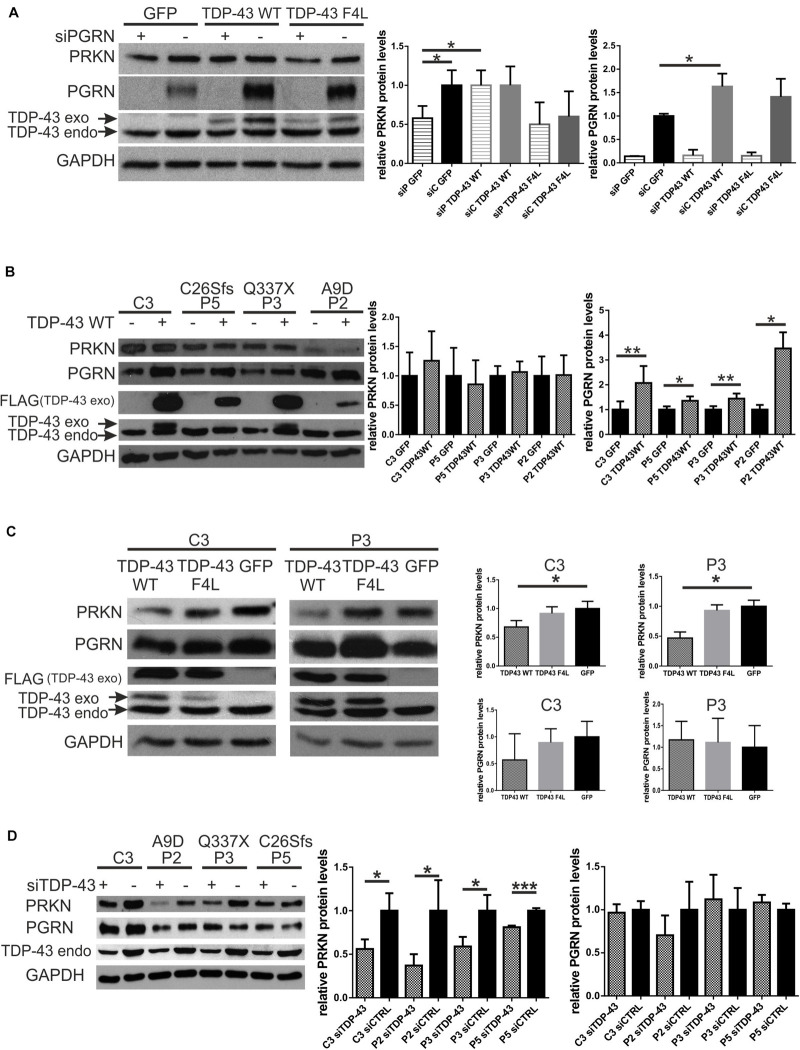
Silencing of TDP-43 further decreases parkin levels in fibroblasts with PGRN pathogenic variants while overexpression of TDP-43 WT can rescue parkin levels only in a model of transient PGRN downregulation. **(A)** PRKN, PGRN, and TDP-43 protein levels were determined by Western blot. PGRN was silenced (designated as “+”) in control fibroblasts and 24 h after transfection with siRNA, cells were transfected with either GFP, Flag-TDP-43 WT or Flag-TDP-43 F4L (an RNA-binding mutant unable to negatively autoregulate the endogenous TDP-43). Cells were harvested after 24 h of plasmids overexpression. Right panels show the relative levels (upon normalization to GAPDH levels) of PRKN and PGRN. The result for scramble siRNA - treated cells, transfected with GFP, was set to 1 and all other experimental variants were normalized to it. *p < 0.05, **p < 0.01; siP, siPGRN; *siC*, siCTRL. **(B)** GFP or Flag-TDP-43 WT (designated as - and +, respectively) were overexpressed for 24 h in fibroblasts with PGRN pathogenic variants and control ones. Fibroblasts with PGRN pathogenic variants derived from FTLD patients are designated as P2, P3, and P5 with respective pathogenic variants marked above: A9D, Q337X, and C26Sfs. Control fibroblast line derived from neurologically healthy individual is designated as C3. 24 h Flag-TDP-43 WT overexpression led to consistent and significant increase of PGRN levels, without the effect on PRKN levels. Right panels show the relative levels (upon normalization to GAPDH levels) of PRKN and PGRN, determined densitometrically. For each individual cell line the result obtained upon GFP-transfection was normalized to 1.*p < 0.05, **p < 0.01; **(C)** Flag-TDP-43 WT or Flag-TDP-43 F4L, or GFP were overexpressed for 48 h in fibroblasts with PGRN mutations and control ones. P3 is a fibroblast line derived from FTLD patient with PGRN mutation Q337X, C3 is a control fibroblast line from neurologically healthy individual. 48 h Flag-TDP-43 WT overexpression led to PRKN downregulation. Right panels represent the relative levels (upon normalization to GAPDH levels) of PRKN and PGRN. The result obtained upon GFP-transfection was normalized to 1, and all other results were normalized to it. *p < 0.05, **p < 0.01; **(D)** TDP-43 was silenced for 48 h (designated as “+”) in fibroblasts with PGRN pathogenic variants and control one. Cells treated with control siRNA (designated as “-”) have been used as a control for each cell line and normalized to 1. Fibroblasts with PGRN pathogenic variants derived from FTLD patients are designated as P2, P3, and P5 with respective amino acid change marked above: A9D, Q337X, and C26Sfs. Control fibroblast line derived from neurologically healthy individual is designated as C3. Right panel shows the relative levels of PRKN and PGRN. *p < 0.05, ***p < 0.001; PRKN, parkin; PGRN, progranulin; TDP-43, transactive response DNA-binding protein 43 kDa; GFP, green fluorescent protein.

In the first model, we silenced *PGRN* in control fibroblasts and 24 h after siRNA administration we transfected GFP, Flag-TDP-43 WT or Flag-TDP-43 F4L plasmids for 24 h. In this model, we observed rescue of parkin upon Flag-TDP-43 WT overexpression but not following Flag-TDP-43 F4L overexpression (Figure 3A). In addition, there was significant upregulation of PGRN by Flag-TDP-43 WT, but not Flag-TDP-43 F4L (Figure 3A). Since the Flag-TDP-43 F4L is a mutant unable to bind RNA (Ayala et al., 2011), these results suggest that the RNA binding function of TDP-43 is crucial both for parkin rescue and PGRN upregulation (Figure 3A).

In the second model, in fibroblasts with PGRN pathogenic variants (A9D, Q337X, and C26Sfs, derived from FTLD patients) and in control fibroblast line (C3), overexpression of Flag-TDP-43 WT for 24 h was not able to upregulate PRKN levels (Figure 3B) while it increased PGRN levels in all tested cell lines (Figure 3B).

However, when we overexpressed Flag-TDP-43 WT for longer times—48 h—we observed downregulation of PRKN levels compared to Flag-TDP-43 F4L mutant or GFP overexpression, both in fibroblast line with PGRN pathogenic variant (Q337X – P3) and in control line (C3) (Figure 3C).

Taken together, our results suggest that the TDP-43 WT overexpression could rescue parkin deficiency only upon transient (Figure 3A) and not life-long PGRN depletion due to the presence of germline *PGRN* pathogenic variants (Figure 3B).

**FIGURE 4:**
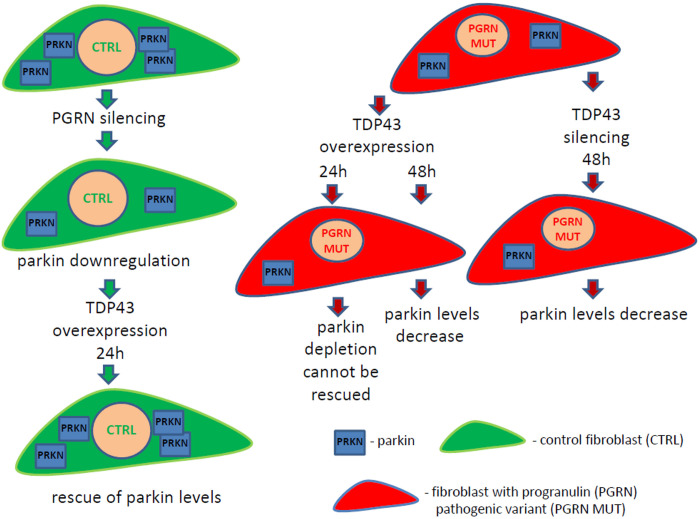
Schematic summary of the obtained results. Fibroblasts with PGRN pathogenic variants from frontotemporal lobar degeneration (FTLD) patients (red color cells) have downregulated parkin protein (blue square “PRKN”) compared to control fibroblasts (green color cells). Transient *PGRN* silencing in control fibroblasts leads to decrease of parkin levels, which can be rescued upon TDP-43 overexpression for 24 h. However, TDP-43 overexpression for 24 h cannot rescue PRKN levels in FTLD fibroblasts with PGRN pathogenic variants, resulting in parkin decrease after 48 h. On the other hand, TDP-43 silencing for 48 h causes parkin downregulation in FTLD fibroblasts with PGRN pathogenic variants. These results suggest that TDP-43 loss-of-function contributes to PRKN decrease. Our data provide further evidence that parkin downregulation might be a common and systemic phenomenon in neurodegenerative diseases with TDP- 43 loss-of-function.

Finally, upon silencing of *TDP-43*, PRKN was downregulated in all analyzed fibroblast cell lines, the control one (C3) and those with PGRN pathogenic variants (P2, P3, and P5), while PGRN levels remained unchanged (Figure 3D). All these experimental interventions and their outcomes are schematically summarized in Figure 4.

## Discussion

The observation of the E3 ligase parkin downregulation in sporadic and various genetic models of TDP-43 proteinopathy (Polymenidou et al., 2011; Lagier-Tourenne et al., 2012; Davis et al., 2018; Palomo et al., 2018) points to TDP-43 as the common denominator and the main culprit of this phenomenon. Indeed, enhanced mitochondrial localization of TDP-43 and its C- and N-terminal fragments indicate that they can interfere with proper mitochondrial functioning (Wang et al., 2016; Davis et al., 2018). In particular, silencing or overexpression of TDP-43 affects the expression or localization of key mitophagy players, such as parkin, PINK1 and PHB2 (Davis et al., 2018).

However, since different genetic backgrounds may modify the final outcome, we addressed the question whether PGRN depletion, inextricably associated with TDP-43 pathology, led to parkin downregulation.

In our work, we have observed a decrease of parkin protein and mRNA levels in fibroblasts derived from carriers of PGRN pathogenic variants (FTLD patients and their asymptomatic younger relatives), compared to controls. This result suggests that parkin depletion occurs early in PGRN deficiency, preceding symptomatic phase. However, this was not accompanied by changes in MFN2 and VDAC1, parkin dowstream targets.

We further confirmed that parkin protein and mRNA levels dropped upon *PGRN* silencing for 48 h (Figures 2A,B and Supplementary Figure 1B), suggesting that the observed parkin decline is due to transcriptional regulation. To date, Palomo et al. (2018) have reported decreased parkin protein with unchanged mRNA levels in a genetic ALS model (mice with G93A *SOD1* pathogenic variant) characterized by TDP-43 proteinopathy.

While upon transient *PGRN* silencing in control, neurologically healthy, age-matched fibroblasts we observed downregulation of parkin partners such as MFN2 and VDAC1 (Figures 2A,B), such changes were not seen in fibroblasts carrying PGRN pathogenic variants (Figure 1A). These differences could be due to compensatory responses to lifelong PGRN deficiency observed in carriers of PGRN pathogenic variants that are absent upon transient *PGRN* silencing in the same cell type, highlighting that there is a limited overlap between the two systems. Also, genetic heterogeneity among tested fibroblast cell lines (each one obtained from a different study participant) could have masked existing differences.

While TDP-43 proteinopathy is observed in the nervous system of FTLD and ALS patients, it has also been characterized in primary fibroblasts derived from ALS patients, both sporadic, and with various causative pathogenic variants (in *C9orf72*, *TARDBP*, *FUS*, and *SOD1* genes) indicating that fibroblast model reflects the changes observed in human brain to some extent (Sabatelli et al., 2015). To confirm the presence of TDP-43 pathology in our fibroblast model, we observed the increased production of the 25 kDa fragment of TDP-43 C-terminal cleavage (Neumann et al., 2006; Zhu et al., 2019) upon silencing of *PGRN* (Figure 2A and Supplementary Figure 2).

To date, parkin depletion has been linked to TDP-43 neuropathology or following manipulations of TDP-43 expression (Polymenidou et al., 2011; Lagier-Tourenne et al., 2012; Hebron et al., 2013; Davis et al., 2018; Sun et al., 2018). The results of TDP-43 depletion have been consistent, leading to parkin mRNA/protein downregulation in different animal and cellular models (Polymenidou et al., 2011; Lagier-Tourenne et al., 2012; Davis et al., 2018). On the other hand, wild-type TDP-43 overexpression has provided discordant results (Sun et al., 2018 vs. Hebron et al., 2013; Davis et al., 2018; see also Supplementary Table 3). Sun et al. (2018) demonstrated parkin downregulation upon overexpression of wild type TDP-43-HA for 48 h in HEK293T cells, and in primary mouse neurons. In addition, knock-in of wild-type human TDP-43 in the Drosophila model caused a significant reduction of the endogenous parkin protein and mRNA. In contrast, other groups demonstrated parkin upregulation upon TDP-43 overexpression in HEK293T and M17 neuroblastoma cells, respectively (Hebron et al., 2013; Davis et al., 2018; Supplementary Table 3).

To better clarify this issue, we both silenced or transiently overexpressed TDP-43 in our two models to see how it affected parkin levels on the background of PGRN haploinsufficiency.

We observed that overexpression of TDP-43 WT for 24 h, increased PGRN levels in all fibroblast lines (Figures 1A,B), likely *via* TDP-43-mediated enhancement of SORT1 expression, specifically, its functional wild type isoform (Prudencio et al., 2012). In parallel, we observed rescue of parkin levels by TDP-43 WT overexpression only in a model of short-time PGRN depletion in control fibroblasts (Figure 3A) but not in fibroblasts with PGRN pathogenic variants (Figure 3B). This may indicate that parkin decrease following short-term silencing of *PGRN* is reversible, in contrast to life-long adaptations to this condition. In addition, TDP-43 F4L mutant, unable to bind mRNA, could not induce nor PGRN (Figures 3A,B) neither parkin upregulation (Figure 3A).

However, TDP-43 WT overexpression for 48 h, but not TDP-43 F4L, in fibroblasts with PGRN pathogenic variants and control ones, led to parkin downregulation (Figure 3C). These results are in concordance with a previous report showing that 48 h TDP-43 overexpression in 293T cell line caused post-transcriptional downregulation of parkin, and it required both the RNA-binding and protein–protein interaction functions of TDP-43 (Sun et al., 2018).

Finally, upon TDP-43 silencing we observed decreased PRKN levels in all fibroblast lines (fibroblasts with PGRN pathogenic variants and control ones) without significantly modifying PGRN levels (Figure 3D). We summarized our results in comparison to published literature in Supplementary Table 3.

Our observation that parkin downregulation occurs both upon TDP-43 overexpression (for 48 h) and silencing may seem apparently contradictory (see Figure 4 and Supplementary Table 3). However, similar modes of regulation have already been described in other biological systems (Wang et al., 2014). For example, both silencing and overexpression of SRPK1 resulted in Akt activation (Wang et al., 2014). It can be speculated that TDP-43 is probably part of a complex that affects parkin expression. If TDP-43 is absent, the complex doesn’t work and parkin expression decreases. On the other hand, when TDP-43 is overexpressed, the stoichiometry of the complex is disrupted and so is its functionality, which would also result in parkin depletion.

In summary, our project, carried out in two models of PGRN haploinsufficiency—fibroblasts from FTLD-PGRN patients and upon silencing of *PGRN* in control fibroblasts (see Figure 4), suggests that TDP-43 loss-of-function contributes to PRKN downregulation. However, we were not able to rescue parkin levels by TDP-43 overexpression in FTLD patient-derived fibroblasts with PGRN mutations (Figures 3B,C). The main limitation of the presented research is that the first and the second model (transient PGRN depletion vs. life-long adaptations to germline PGRN mutations, respectively) cannot be directly compared due to before mentioned inherent differences.

In addition, our results indicate that parkin decrease associated with PGRN depletion reflects a systemic event, present in tissues not affected by degeneration, such as skin fibroblasts. This finding underlines the need to analyze the extend of parkin downregulation in neuronal and neuroglial models of PGRN deficiency along with its potential functional effects on mitophagy and other mitochondrial functions in the nervous system. In particular, it would be interesting to compare the consequences of parkin dysfunction between PD caused by *PARK2* pathogenic variants (Mortiboys et al., 2008) and FTLD/ALS models with TDP-43 pathology.

On the other hand, it is known from various animal/cellular models that PGRN deficiency leads to upregulation of lysosomal machinery, responsible for clearance of PINK1/parkin-primed mitochondria (Evers et al., 2017; Tanaka et al., 2017; Götzl et al., 2018). Interestingly, there are lines of evidence suggesting that parkin downregulation could contribute to this phenomenon, as p62 levels (an autophagic marker) are controlled by parkin and they increase in brains of parkin knockout mice (Song et al., 2016). Further investigation of these interconnections will explain how decreased parkin levels could contribute to already reported dysfunctional lysosomal phenotype associated with PGRN deficiency (Tanaka et al., 2017; Paushter et al., 2018).

## Data Availability Statement

The raw data supporting the conclusions of this article will be made available by the authors, without undue reservation.

## Ethics Statement

The studies involving human participants were reviewed and approved by Bioethical Committee of the Central Clinical Hospital of the Ministry of Interior Affairs and Administration in Warsaw and the Bioethical Committee of Medical University of Gdańsk. The patients/participants provided their written informed consent to participate in this study.

## Author Contributions

KG-W: conceptualization, methodology, project administration, supervision, and writing of original draft, review and editing. KG-W, EB, and DW: formal analysis. KG-W, DW, and MB: validation and investigation. CZ, DW, and MB: funding acquisition. KG-W, DW, CZ, MB, and EB: resources. KG-W, EB, DW, and CZ: writing – review and editing. All authors contributed to the article and approved the submitted version.

## Conflict of Interest

The authors declare that the research was conducted in the absence of any commercial or financial relationships that could be construed as a potential conflict of interest.
