# Supplementary material for: Parkin Levels Decrease in Fibroblasts With Progranulin (PGRN) Pathogenic Variants and in a Cellular Model of PGRN Deficiency
Source: Front Mol Neurosci. 2021 May 13;14:676478. doi: 10.3389/fnmol.2021.676478 (PMC8155584; doi:10.3389/fnmol.2021.676478)
Supplement: Supplementary file 1 [file Data_Sheet_1.pdf]

1 Supplementary Data

Table S1. Subjects who participated in the study

Name		PGRN variant (aa change)	diagnosis	reference	rs
P1*	unrelated	M1L	FTLD	(Chen-Plotkin et al. 2011)	rs746037872
P2*	unrelated	A9D	FTLD	(Spina et al. 2007; Mukherjee et al. 2008; Cannon et al. 2013)	rs63751243
P3	Family 1	Q337X	FTLD	(Chen-Plotkin et al. 2011)	rs1598364961
A4	Family 1	Q337X	asymptomatic	(Chen-Plotkin et al. 2011)	rs1598364961
Control 1 (C1)	Family 1	-	neurologically healthy		-
P5	Family 2	C26Sfs	FTLD	(Gaweda-Walerych et al. 2018)	na/absent in ExAC,1000gp, ESP5400 databases

A6	Family 2	C26Sfs	asymptomatic	(Gaweda-Walerych et al. 2018)	na/ absent in ExAC,1000gp, ESP5400 databases
Control 2 (C2)	Family 2	-	neurologically healthy	(Gaweda-Walerych et al. 2018)	-
Control 3 (C3)	unrelated	-	neurologically healthy	(Gaweda-Walerych et al. 2018)	-
Control 4 (C4)	unrelated	-	neurologically healthy	(Gaweda-Walerych et al. 2018)	-

Gender/age of patients and family members are omitted for confidentiality; Gender/age of control 3 is F/55, and of control 4 is M/65, F- female; M- male; FTLD - Frontotemporal lobar degeneration;

*- P1 and P2 cell lines were obtained through the NINDS Human Cell and Data Repository (NHCDR) at Infinity Biologix (formerly RUCDR; NHCDR IDs: ND42493 and ND40082, respectively)

The presence of *PGRN* variants in fibroblast lines C26Ssf and Q337X (P3, A4, P5, A6) have been confirmed by Sanger sequencing in our laboratory. Four carriers of the two following mutations: Q337R, C26sf, have been tested in addition for the presence of *C9orf72* expansion, and mutations in *MAPT* gene, since variants in these two genes are the most common cause of FTLD, apart from *PGRN* variants. Methodology for the detection of *C9orf72* expansion and variants in *PGRN* and *MAPT* genes have been described previously (Gaweda-Walerych et al. 2018).

aa – amino acid

rs number – annotation number in dbSNP database

na - not available

Table S2. Primers used in the study

Gene	Primer sequences	source
PGRN	TTGCTGCTGCCCAAGGAC<br>GCCATTTGTCCAGAAGGGGA	(Gaweda-Walerych et al. 2018)
GAPDH	GTTCGACAGTCAGCCGCATC<br>GGAATTTGCCATGGGTGGA	(Gaweda-Walerych et al. 2016; Chung et al. 2017)
PARK2	CCACACTGCCCTGGGACTA<br>TTGTTGCGATCAGGTGCAAA	Designed for this study (NCBI primer blast)
PPIB	GGTGATCTTTGGTCTCTTCGG<br>TAGATGCTCTTTCCTCCTGTG	(Lefever et al. 2009)
MFN2	CTGTCTGGGACCTTTGCTCA<br>TTGCGCTCCAGCAAATGAAC	Designed for this study (NCBI primer blast)
VDAC1	GCCCGGAAGGCAGAAGAT<br>GGTGGTCTCAGTGTTGGCTG	Designed for this study (NCBI primer blast)

Table S3. TDP-43 overexpression or depletion can lead to parkin downregulation. Overview of the literature data and the results obtained in this manuscript.

Manipulation type	Vector/time	Cell type /animal model	outcome	reference
TDP-43 knockdown	antisense oligonucleotides injection (TDP-43-specific or control) /2 weeks	mouse adult brain (the striatum of 8–10 week old female C57Bl/6)	decreased parkin mRNA	(Polymenidou et al. 2011)
TDP-43 knockdown	lentiviral shRNA constructs; TDP-43- or GFP- specific (control)/na	human neurons (HUES6 line and iPSC -derived)	decreased parkin mRNA	(Lagier-Tourenne et al. 2012)
overexpression	wild type TDP-43-HA/ 48 h	human HEK293T cells	decreased parkin mRNA	(Sun et al. 2018)
overexpression	wild-type TDP-43-HA/ 48 h	primary mouse neurons	decreased parkin mRNA	(Sun et al. 2018)
knock-in	wild-type human TDP-43-H/na	Drosophila model	decreased parkin protein and mRNA	(Sun et al. 2018)
overexpression	wild type pLX-TDP-43-v5 vector/na	human HEK293T cells (DMSO vs. mitochondrial uncoupler CCCP)	increased parkin cytoplasmic localization	(Davis et al. 2018)
silencing of TDP43	siRNA against TDP-43 or siRNA CTRL/na	human HEK293T cells (DMSO vs. mitochondrial uncoupler CCCP)	decreased parkin cytoplasmic localization	(Davis et al. 2018)
TDP-43 overexpression	cDNA transfection (WT TDP-43 or control) /24h	human M17 neuroblastoma cells	increased parkin protein	(Hebron et al. 2013)

overexpression	wild type flag-TDP-43/24h	human primary skin fibroblasts with transiently silenced PGRN	increased parkin protein	this manuscript
overexpression	wild type flag-TDP-43/24h	human primary skin fibroblasts with/without PGRN mutations	no significant difference	this manuscript
overexpression	wild type flag-TDP-43/48h	human primary skin fibroblasts with/without PGRN mutations	decreased parkin protein	this manuscript
silencing of TDP43	siRNA against TDP-43 or siRNA CTRL)/48h	human primary skin fibroblasts with/without PGRN mutations	decreased parkin protein	this manuscript

na – not available

Please, note, that different conditions and methodology could contribute to different outcomes of experiments. E.g. PGRN expression levels are known to be sensitive to experimental procedures. For example, DMSO (used by Davis et al.2018) is known to induce PGRN expression levels (Ong et al. 2006; Raitano et al. 2015). Likewise, viral shRNA has been reported to induce PGRN levels (Rosen et al. 2011). Elevated basal PGRN levels could affect TDP-43 function and in consequence parkin levels.

Fig. S1

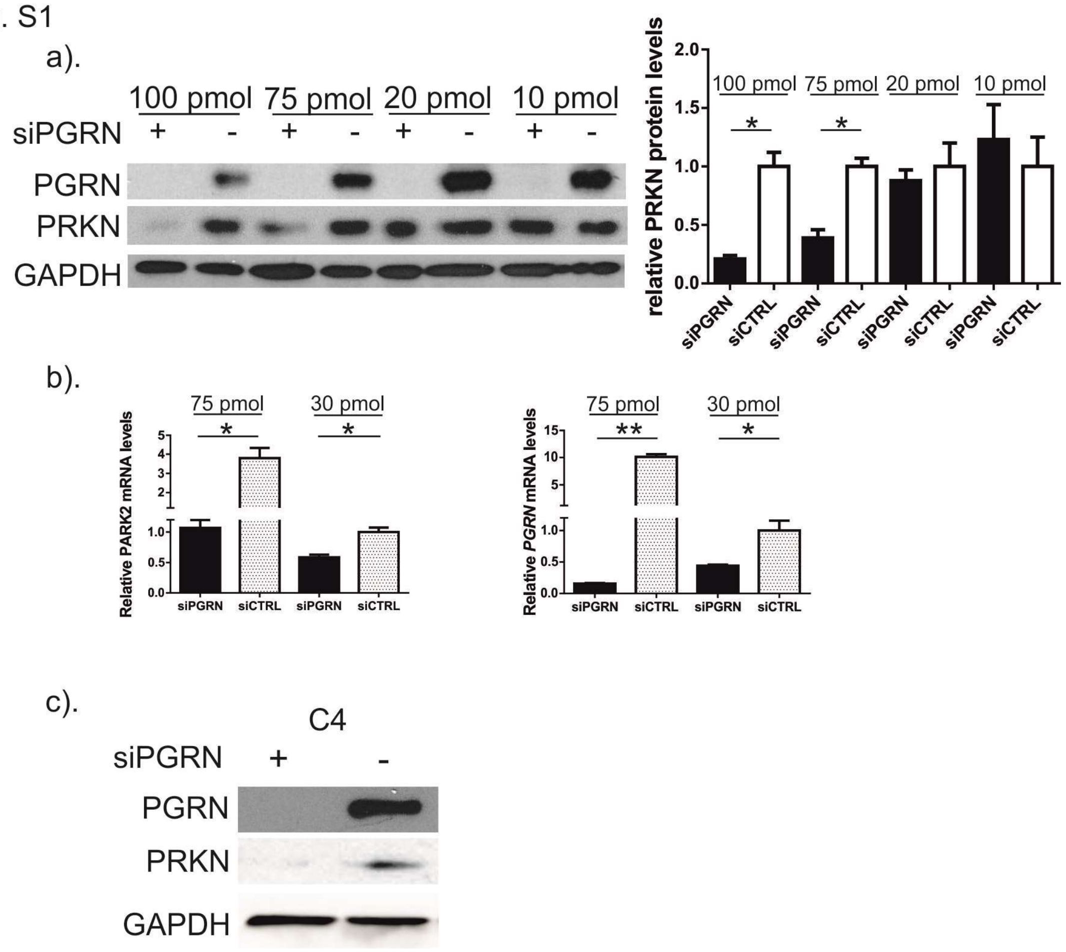

Fig S1 Parkin levels are downregulated in control fibroblasts upon different doses of PGRN siRNA

A). PRKN and PGRN protein levels were determined by Western blot upon *PGRN* silencing (100, 75, 20, and 10 pmol of *PGRN* siRNA vs siRNA scramble) in control fibroblast line C3 cultured in glucose (GLC) medium for 72h; GAPDH was used as a protein load control. Right panel shows densitometric measurements of PRKN and PGRN protein levels (normalized to GAPDH levels); $*p < 0.05$;

B). *PRKN* and *PGRN* mRNA levels were determined by real-time PCR upon *PGRN* silencing (75 and 30 pmol of *PGRN* siRNA or siRNA scramble) in control fibroblast line C3 cultured in glucose (GLC) medium for 48h; *PPIB* was used as reference gene.

C). PRKN and PGRN protein levels were determined by Western blot upon *PGRN* silencing for 48h in control fibroblast line C4 cultured in glucose (GLC) medium; GAPDH was used as a protein load control.

Fig. S2

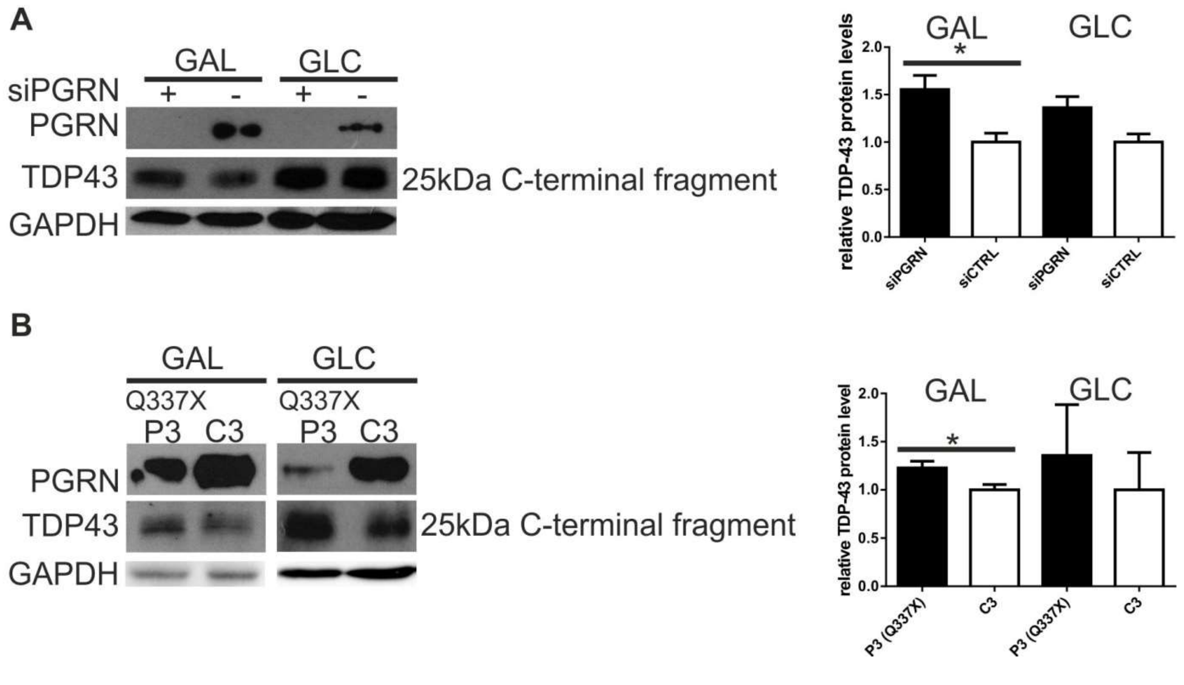

Fig S2. Increased production of the 25kDa fragment derived from TDP-43 C-terminal cleavage in models of PGRN deficiency.

(A) The protein level of TDP-43 C-terminal cleavage fragment of 25kDa was determined by Western blot upon *PGRN* silencing for 72h, compared to scramble siRNA, in control fibroblast line C3 cultured in glucose (GLC) or galactose (GAL) medium; GAPDH was used as a protein load control. Right panel shows densitometric measurements of TDP-43 25kDa C-terminal product levels (normalized to GAPDH level); $*p < 0.05$,

(B) The protein level of TDP-43 C-terminal cleavage fragment of 25kDa was determined by Western blot in fibroblasts derived from FTLD patient (P3) with PGRN pathogenic variant Q337X, compared to control fibroblast line (C3). Fibroblasts were cultured on galactose (GAL) and glucose (GLC) medium for 72h; GAPDH was used as a protein loading control. Right panel shows densitometric measurements of TDP-43 25kDa C-terminal product levels (normalized to GAPDH level); $*p < 0.05$,

Abbreviations: PRKN- parkin; PGRN – progranulin, TDP-43 - transactive response DNA-binding protein 43 kDa).

References:

Cannon, A., et al. (2013). "Clinicopathologic variability of the GRN A9D mutation, including amyotrophic lateral sclerosis." Neurology.

Chen-Plotkin, A. S., et al. (2011). "Genetic and clinical features of progranulin-associated frontotemporal lobar degeneration." Arch Neurol.

Chung, S. W., et al. (2017). "Altered Gene and Protein Expressions in Torn Rotator Cuff Tendon Tissues in Diabetic Patients." Arthroscopy.

Davis, S. A., et al. (2018). "TDP-43 interacts with mitochondrial proteins critical for mitophagy and mitochondrial dynamics." Neurosci Lett.

Gaweda-Walerych, K., et al. (2016). "Parkinson's disease-related gene variants influence pre-mRNA splicing processes." Neurobiol Aging.

Gaweda-Walerych, K., et al. (2018). "Functional characterization of a novel progranulin mutation in a patient with progressive nonfluent aphasia." Neurobiol Aging.

Hebron, M. L., et al. (2013). "Parkin ubiquitinates Tar-DNA binding protein-43 (TDP-43) and promotes its cytosolic accumulation via interaction with histone deacetylase 6 (HDAC6)." J Biol Chem.

Lagier-Tourenne, C., et al. (2012). "Divergent roles of ALS-linked proteins FUS/TLS and TDP-43 intersect in processing long pre-mRNAs." Nat Neurosci.

Lefever, S., et al. (2009). "RTPrimerDB: the portal for real-time PCR primers and probes." Nucleic Acids Res.

Mukherjee, O., et al. (2008). "Molecular characterization of novel progranulin (GRN) mutations in frontotemporal dementia." Hum Mutat.

Ong, C. H., et al. (2006). "Regulation of progranulin expression in myeloid cells." Am J Physiol Regul Integr Comp Physiol.

Polymenidou, M., et al. (2011). "Long pre-mRNA depletion and RNA missplicing contribute to neuronal vulnerability from loss of TDP-43." Nat Neurosci.

Raitano, S., et al. (2015). "Restoration of progranulin expression rescues cortical neuron generation in an induced pluripotent stem cell model of frontotemporal dementia." Stem Cell Reports.

Rosen, E. Y., et al. (2011). "Functional genomic analyses identify pathways dysregulated by progranulin deficiency, implicating Wnt signaling." Neuron.

Spina, S., et al. (2007). "Corticobasal syndrome associated with the A9D Progranulin mutation." J Neuropathol Exp Neurol.

Sun, X., et al. (2018). "Distinct multilevel misregulations of Parkin and PINK1 revealed in cell and animal models of TDP-43 proteinopathy." Cell Death Dis.
